# Temporal activation of XRCC1-mediated DNA repair is essential for muscle differentiation

**DOI:** 10.1038/celldisc.2015.41

**Published:** 2016-01-12

**Authors:** Mohammad H Al-Khalaf, Leanne E Blake, Brian D Larsen, Ryan A Bell, Steve Brunette, Robin J Parks, Michael A Rudnicki, Peter J McKinnon, F Jeffrey Dilworth, Lynn A Megeney

**Affiliations:** 1 Sprott Centre for Stem Cell Research, Regenerative Medicine Program, Ottawa Hospital Research Institute, The Ottawa Hospital, Ottawa, ON, Canada; 2 Department of Cellular and Molecular Medicine, Faculty of Medicine, University of Ottawa, Ottawa, ON, Canada; 3 Department of Genetics, St Jude Children's Research Hospital, Memphis, TN, USA

**Keywords:** base excision repair, XRCC1, muscle differentiation, DNA strand breaks

## Abstract

Transient DNA strand break formation has been identified as an effective means to enhance gene expression in living cells. In the muscle lineage, cell differentiation is contingent upon the induction of caspase-mediated DNA strand breaks, which act to establish the terminal gene expression program. This coordinated DNA nicking is rapidly resolved, suggesting that myoblasts may deploy DNA repair machinery to stabilize the genome and entrench the differentiated phenotype. Here, we identify the base excision repair pathway component XRCC1 as an indispensable mediator of muscle differentiation. Caspase-triggered XRCC1 repair foci form rapidly within differentiating myonuclei, and then dissipate as the maturation program proceeds. Skeletal myoblast deletion of *Xrcc1* does not have an impact on cell growth, yet leads to perinatal lethality, with sustained DNA damage and impaired myofiber development. Together, these results demonstrate that XRCC1 manages a temporally responsive DNA repair process to advance the muscle differentiation program.

## Introduction

Genome stability is of utmost importance for the survival and development of all organisms. Accordingly, eukaryotic cells have evolved a variety of mechanisms to maintain and repair DNA, of which these mechanisms are collectively referred to as the DNA damage response (DDR) [1, 2]. The DDR is further delineated by the type of DNA damage that is targeted and repaired, which may involve single [3] or double-strand breaks [4] and formation of toxic DNA adducts [5]. Although persistent DNA damage is generally regarded as detrimental for maintaining cell viability, transient DNA strand break formation has been identified as an effective means to enhance gene expression in living cells [6–9]. The best studied example in this regard is V(D)J recombination, a purposeful DNA damage and strand rearrangement event that underlies the genetic diversity of antibody and T-cell receptor generation in the adaptive immune system [10].

More recently, DNA strand breakage has been demonstrated to exert a profound effect on cell fate, acting to limit stem cell self-renewal and stimulate differentiation, without a concomitant increase in cell death [11–19]. In the skeletal muscle lineage, cell differentiation is dependent upon a temporal activation of the caspase 3 protease and its cognate DNase CAD (caspase-activated DNase), which act to enhance muscle gene expression through targeted DNA strand breaks [11]. These observations imply that a differentiating cell must utilize/recruit an equally potent DNA repair mechanism to stabilize the genome and secure the cell fate selection. The corollary to this hypothesis predicts that loss or inhibition of such a repair mechanism would result in failure to establish the differentiated state. Here, we sought to identify the repair machinery/proteins that target differentiation-induced DNA strand breaks and whether this operant mechanism was similar to or divergent from existing DNA damage responses (DDRs). Our observations indicate that base excision-mediated repair, as exemplified by the scaffold protein XRCC1, is essential for resolving differentiation-associated DNA damage and for securing this cell fate choice.

## Results

### The base excision repair pathway is involved in early myoblast differentiation

**Figure 1:**
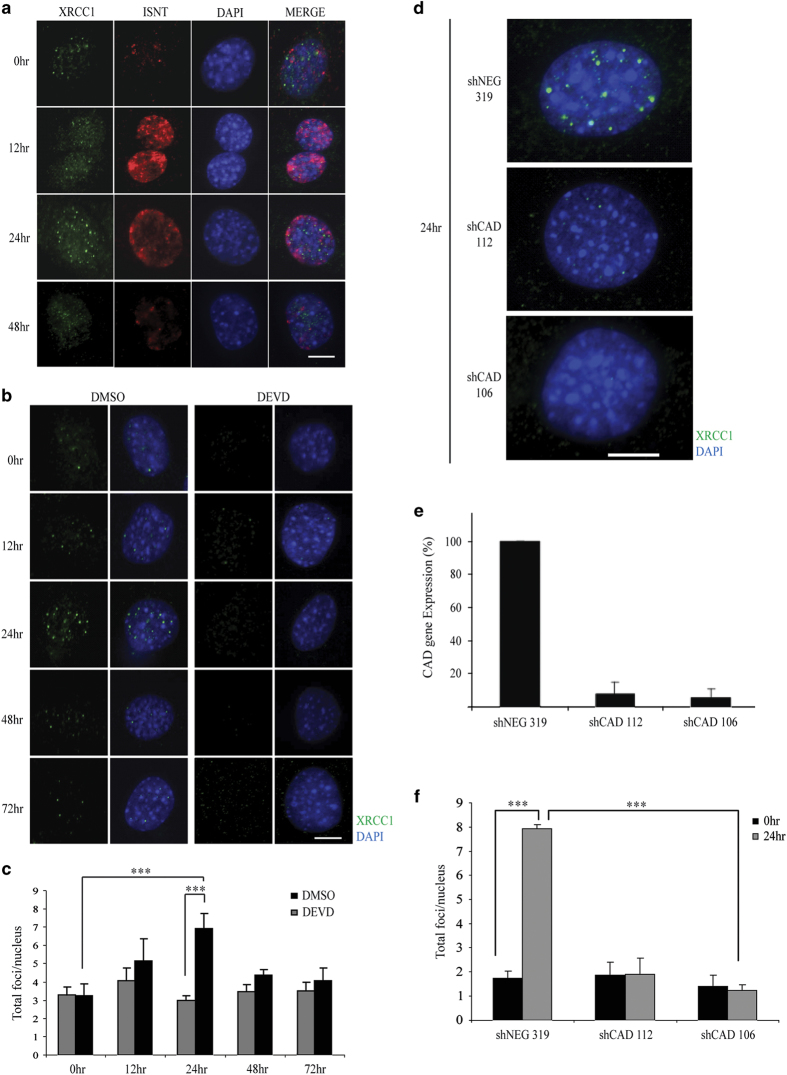
DNA repair during early myoblast differentiation is associated with XRCC1 foci. (**a**) Co-staining for XRCC1 foci formation and *in situ* nick translation, to measure DNA polymerase activity, in C2C12 myoblast cells over differentiation time course. Scale bar, 10 μm. (**b**) Immunofluorescent staining for XRCC1 in caspase 3 inhibited (DEVD) differentiating C2C12 cells and counterstained using 4′,6-Diamidino-2-phenylindole dihydrochloride (DAPI). Scale bar, 10 μm. Images are representative from *n*=3 experimental replicates. (**c**) Data were quantified by counting the total number of foci per nucleus as represented in the histogram. (**d**) Targeted shRNA-mediated knockdown of CAD in differentiating C2C12 cells. The cells were induced to differentiate for 24 h and then were immunofluorescently stained for XRCC1. (**e**) Quantitative real-time PCR reveals an 80% reduction in CAD expression in the knockdown lines compared with the control. (**f**) Quantification of total XRCC1 foci per nucleus in proliferating cells (growth media) and after 24-h differentiation (24 h) is represented by histogram (*n*=3). Asterisks (***) indicate that the changes in foci formation between the two conditions indicated are statistically significant as determined by two-tailed Student's *t*-test analysis with *P*-value<0.01.

Skeletal muscle cells cultured under low-serum conditions recapitulate the differentiation program and are characterized by transient strand break formation [11]. Using this model system, we noted that the standard components of the nonhomologous end joining (NHEJ) pathway were not active during differentiation, as muscle cells did not display increased foci formation for the ATM kinase, 53BP1 and Ligase IV (Supplementary Figure S1a). Differentiating muscle cells give rise to long-lived cell types; therefore, we reasoned that the DNA damage may be more reflective of single-strand breaks/nicks versus catastrophic double-strand breaks. In support of this supposition, we noted that XRCC1, a key scaffold protein in the base excision repair (BER) pathway [14, 20, 21], forms discrete foci during differentiation (Figure 1a and Supplementary Figure S1a). The XRCC1 foci are transient and match the temporal DNA strand breaks that form during muscle cell differentiation (Figure 1a,b and c; as measured by DNA polymerase-guided *in situ* nick translation (INST)). Next, we ascertained whether these XRCC1 foci resulted from the caspase 3/CAD-mediated strand break activity that characterizes muscle cell differentiation [11, 22]. Using short hairpin RNA (shRNA) CAD muscle cell lines (previously described by our group [11]), we noted that loss of CAD expression resulted in a complete absence of XRCC1 foci formation (Figure 1d and e). Moreover, peptide inhibition of caspase 3 activity during muscle cell differentiation results in a complete loss of XRCC1 foci formation (Figure 1f). These results confirm that XRCC1 clustering in differentiating cells is a direct response to the caspase 3/CAD-induced DNA damage. These results also establish that this DNA break/replication repair cycle is a differentiation-specific event rather than a terminal mitosis response.

### XRCC1 deletion halts C2C12 myoblast fusion and myotube formation

**Figure 2:**
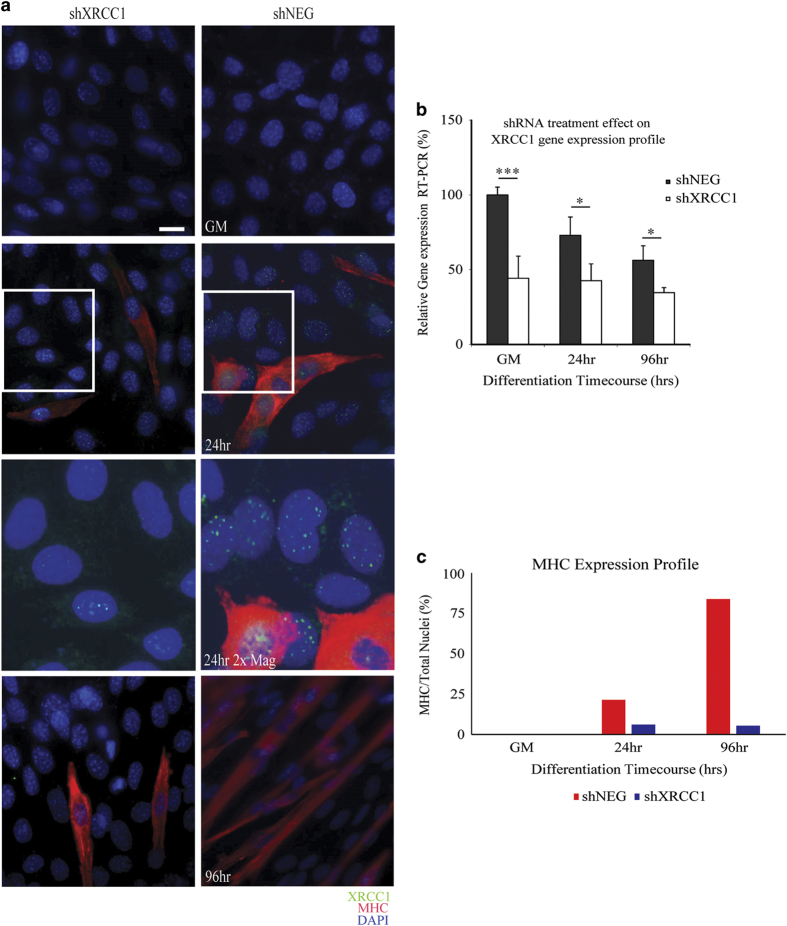
shRNA-mediated loss of *Xrcc1* impedes myoblast differentiation. (**a**) Differentiation time course of shRNA-treated C2C12 myoblasts. Magnified inset panels included for 24-h differentiated conditions performed using the Photoshop CS3 software (Adobe Systems Inc., San Jose, CA, USA). (**b**) Real-time PCR demonstrates a reduction in *Xrcc1* gene expression by 50% in shRNA-transfected cells at growth (*n*=3, one-tailed Student's *t*-test analysis with ***P-value<0.01 and *P<0.05). (**c**) Scoring the percentage of differentiated C2C12 cells (myosin heavy chain (MHC) cells/total nuclei) shows a decrease in differentiation after loss of XRCC1. Images are representative from *n*=3 experimental replicates. Scale bar, 50 μm.

To assess the impact of XRCC1 expression in differentiating muscle cells, we initially performed shRNA-targeted *Xrcc1* (shXRCC1) gene repression. C2C12 muscle cells were co-transfected with shXRCC1/dsRED plasmid or negative control shRNA (shNEG) and dsRED immediately before low-serum induction of differentiation (Figure 2a and Supplementary Figure S2a). At 96 h post-low-serum exposure, shXRCC1-transfected muscle cells displayed significant impairment in the ability to form multinucleate myotubes with a concurrent reduction in the expression of differentiation-specific proteins (myosin heavy chain; Figure 2c and Supplementary Figure S2c). At all time points of equal transfection with shNEG or shXRCC1 (Supplementary Figure S2b), we noted a significant reduction in *Xrcc1* gene expression (Figure 2b), as well as XRCC1 protein foci (Figure 2a).

### *In vivo* deletion of the *Xrcc1* gene during early differentiation inhibits muscle tissue development

**Figure 3:**
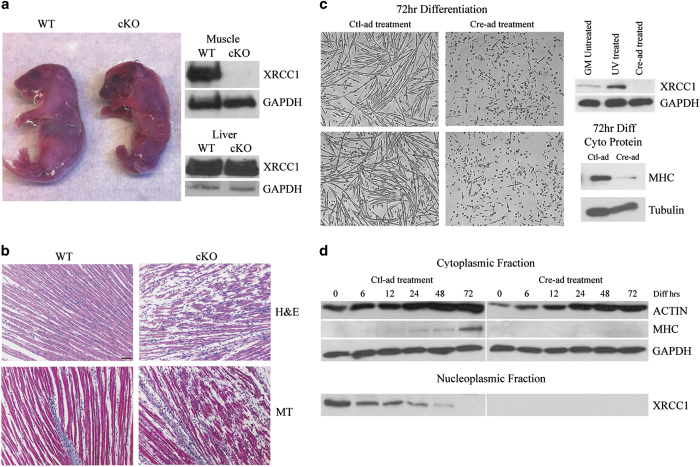
Gene-targeted loss of XRCC1 leads to attenuation of myofiber development and perinatal lethality. (**a**) Wild-type (WT) and *Myf5*-*Cre*/ *Xrcc1*^flox/flox^ conditional knockout (cKO) of *Xrcc1* pup images taken immediately post birth. Nuclear protein extraction from pooled hindleg muscles, and from liver as control, is used for western blot and probed for XRCC1 and GAPDH. Images representative from *n*=5 for each genotype. Western blot is representative from *n*=3 per genotype. (**b**) Longitudinal skeletal muscle sections stained for hematoxylin and eosin (H&E) or Masson’s Trichrome. Images representative from *n*=3. Scale bar, 200 μm. (**c**) Primary myoblasts from *Xrcc1*^flox/flox^ mice are treated with Cre-ad or Negative Ctl-ad and then induced to differentiate for 72 h. Images representative from *n*=5. Scale bar, 200 μm. Western blot probed for XRCC1 is from nuclear protein fraction isolated from *Xrcc1*^flox/flox^ primary myoblasts, either untreated, ultraviolet-treated to induce DNA damage or Cre-ad-treated to delete the *Xrcc1* gene. Western blot is representative from *n*=3. Western blot for myosin heavy chain (MF20) in cytoplasmic protein lysates from *Xrcc1*^flox/flox^ primary myoblasts that were treated with either Cre-ad or Ctl-ad, and induced to differentiate for 72 h. Western blot is representative from *n*=3. (**d**) Western blot analysis from a time course treatment of *Xrcc1*^flox/flox^ primary myoblasts induced to differentiate by low-serum exposure. Blots representative from *n*=3.

Next, we sought to assess the consequences of *in vivo* disruption of XRCC1 expression on skeletal muscle cell differentiation and muscle fiber maturation. *Xrcc1* null mice are early embryonic lethal [23]; as such, we generated a skeletal muscle conditional *Xrcc1* knockout model by cross-breeding *Xrcc1*^flox/flox^ mice [24] with the *Myf5*-*Cre* mouse strain [25, 26]. *Myf5*-*Cre*/ *Xrcc1*^flox/flox^ mice were born in normal Mendelian ratios (Supplementary Figure S3c and d), yet all these animals suffered early perinatal lethality (Figure 3a and Supplementary Figure S4a). Nuclear protein extracted from pooled hindleg muscle showed a marked decrease in XRCC1 expression in the *Myf5*-*Cre*/ *Xrcc1*^flox/flox^ mice relative to non-*Cre Xrcc1* controls (Figure 3a and Supplementary Figure S3a and b). This was specific to muscle, as tissue collected from non-*Cre*-targeted organs, such as the liver, did not show any marked change in XRCC1 protein expression (Figure 3a). *Myf5*-*Cre*/ *Xrcc1*^flox/flox^ mice (body weight=1.10 ±0.1 g, *n*=5) were significantly smaller by 35% than wild-type littermates (body weight=1.70 ±0.2 g, *n*=5) and displayed a severe lack of skeletal muscle development. Histologic examination of *Myf5*-*Cre*/ *Xrcc1*^flox/flox^ skeletal muscle revealed blunted myofiber formation, increased prevalence of mononucleated muscle cells and increased interstitial space (Figure 3b and Supplementary Figure S4b)—changes that are consistent with a severe perturbation in the muscle cell maturation program. To confirm that the *Myf5*-*Cre*/ *Xrcc1*^flox/flox^ skeletal muscle phenotype arose from a differentiation deficit, we examined differentiation kinetics in primary myoblasts isolated from *Xrcc1*^flox/flox^ mice. *Cre*-adenovirus (Cre-ad)-infected *Xrcc1*^flox/flox^ myoblasts displayed a thorough inhibition of the differentiation program, characterized by a loss in expression of myosin heavy chain and a complete inability to form multinucleate myotubes compared with control-Adenovirus (Ctl-ad)-infected cells (Figure 3c and d and Supplementary Figure S1b and c).

### Loss of XRCC1 affects late muscle differentiation-specific and cell cycle-specific genes

**Figure 4:**
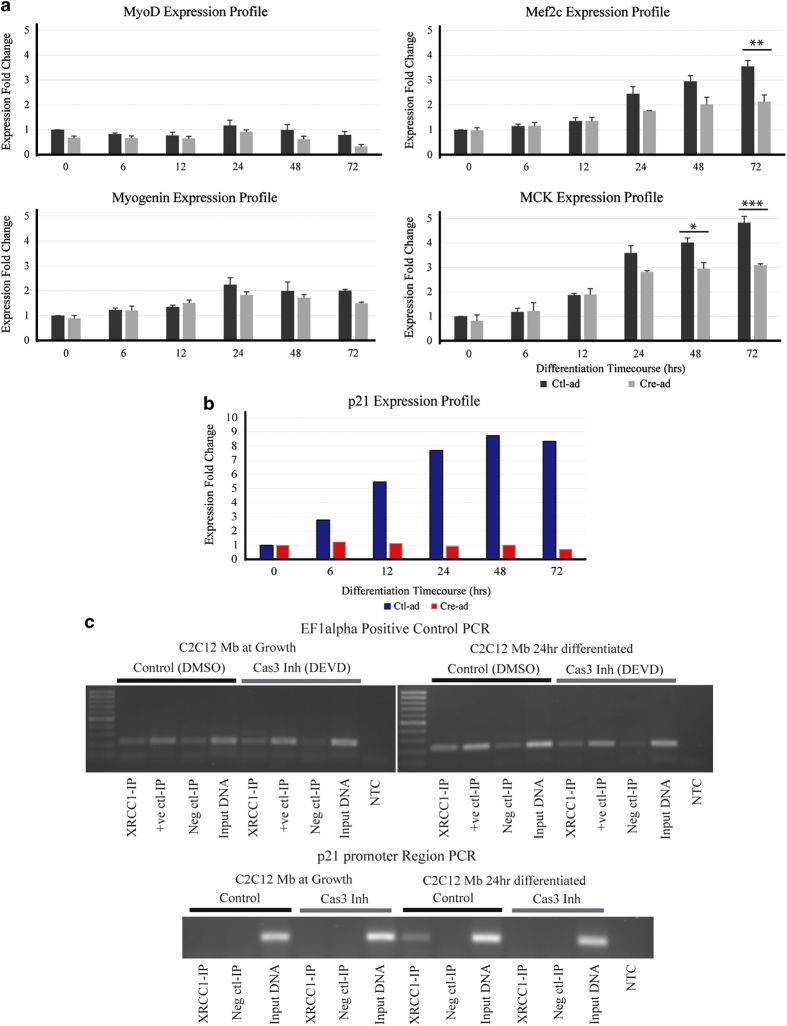
Skeletal muscle gene expression profiles are altered with *XRCC1* deletion. (**a**) Muscle-specific gene markers *myoD*, *myogenin* are unaffected by XRCC1 expression, whereas *Mef2c* and *MCK* inductions are significantly reduced in XRCC1-deleted cells (*n*=3, two-tailed Student's *t*-test analysis with ***P-value<0.01, **P-value<0.025, *P-value<0.05). (**b**) *p21* gene expression profile during early differentiation. *Xrcc1*^flox/flox^ primary myoblasts infected with Ctl-ad (blue) or Cre-ad (red) and induced to differentiate up to 72 h displayed reduced p21 induction (**c**) ChIP-end point PCR of the p21 promoter region shows enhancement of XRCC1 binding during early differentiation of C2C12 cells compared with growth conditions (*n*=3). Caspase 3 inhibition (DEVD) leads to loss of XRCC1-p21 promoter binding. EF1alpha is used as a non-target genomic control for the ChIP experiment.

The shRNA depletion and the *Cre*-mediated excision of the *Xrcc1* gene strongly suggest that muscle cell differentiation is dependent on engaging a temporally sensitive XRCC1-mediated DNA repair event. Real-time PCR for canonical skeletal muscle differentiation markers was performed on control and *Xrcc1*-deleted primary myoblasts, and the results show that, while early-differentiation markers *myoD* and *myogenin* were not significantly altered by the loss of XRCC1, late-differentiation markers *Mef2c* and *MCK* were significantly reduced compared with control conditions (Figure 4a). In addition to muscle-specific genes, prior observations from our laboratory have established that caspase/CAD-induced DNA strand breaks act as a priming event to engage gene expression of nonlineage-specific regulatory factors. For example, a CAD-induced strand break in the promoter region of the cyclin-dependent kinase inhibitor 1 (p21) leads to induction of *p21* expression [11], a gene coding a protein critical for induction of differentiation across a broad spectrum of cell types [27]. Here, using chromatin immunoprecipitation-polymerase chain reaction (ChIP-PCR), we show that XRCC1 binds directly on the same *p21* promoter region upon induction of differentiation to repair the single-strand break (SSB)-induced by CAD (Figure 4c). When XRCC1 is absent, the *p21* gene is not transcribed, as we are able to show using Cre-ad infection of primary *Xrcc1* null myoblast cultures, leading to a complete failure to induce *p21* expression during differentiation (Figure 4b), suggesting that the post-strand break repair event is critical for gene induction at this loci.

### Temporal activation of the *Xrcc1* gene is responsible for progression of myoblast differentiation

**Figure 5:**
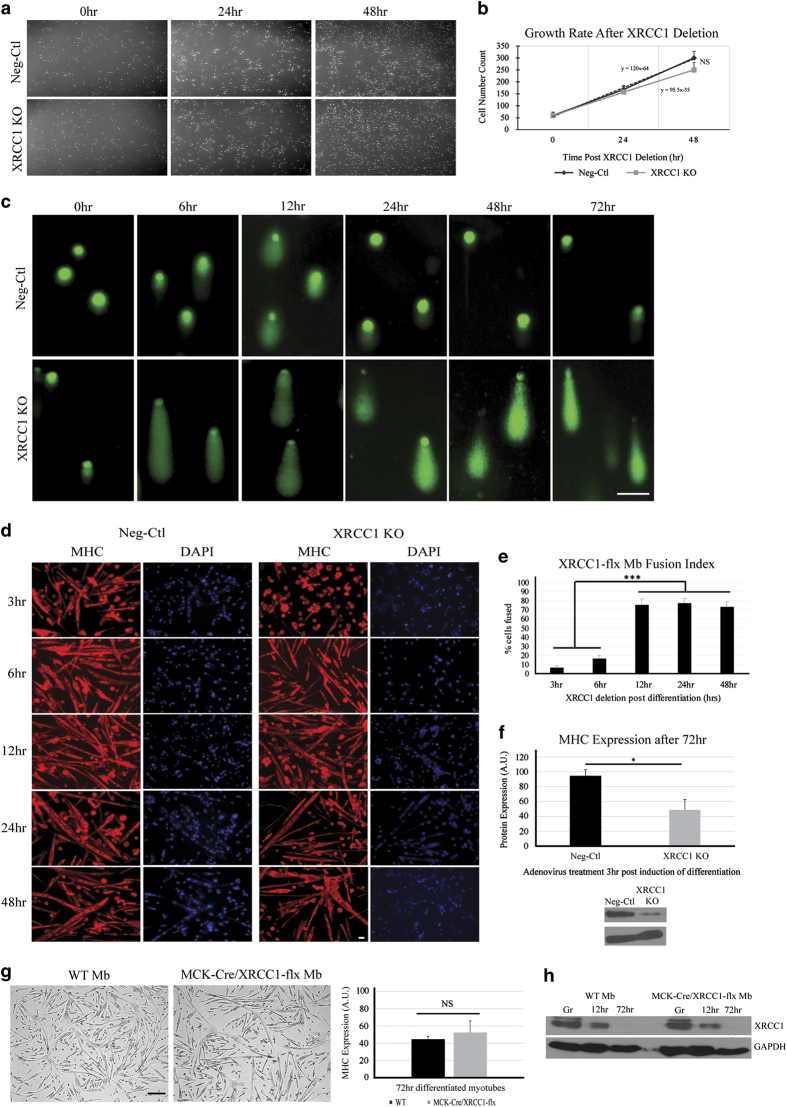
XRCC1 has a temporally sensitive requirement to mitigate DNA damage in differentiating myoblasts. (**a**) Knockout of *Xrcc1* gene expression does not significantly affect myoblast proliferation potential. (**b**) Cell number counts quantified in graph. Images are representative from *n*=6. (**c**) Single-cell gel electrophoresis (Comet assay) was performed on differentiating *Xrcc1*^flox/flox^ primary myoblast cells treated with either Cre-ad or Ctl-ad. Following electrophoresis, cells were stained with SYBR green and visualized to assess the length of migration of DNA from the nucleus. Results show increased and persistent DNA damage in *Xrcc1* knockdown cells, whereas control cells exhibit damage early in differentiation that is resolved over the differentiation time course. Images are representative from *n*=4 for each experimental condition; minimum number of cells counted =50 for each condition. Scale bar, 15 μm. (**d**) Cre-ad-mediated knockdown of *Xrcc1* gene expression post induction of differentiation. Immunofluorescence staining for MHC was used to assess myotube formation. (**e**) The fusion Index graph quantifies myotube formation (*n*=3, two-tailed Student's *t*-test analysis with ***P-value<0.01). Scale bar, 20 μm. (**f**) Quantification of MHC protein at 3 h post induction of differentiation following adenovirus treatment. End point is 72 h post induction of differentiation (*n*=3, two-tailed Student's *t*-test analysis with *P-value<0.05). (**g**) Primary myoblasts isolated from *MCK*-*Cre*/*Xrcc1*^flox/flox^ transgenic mice show equivalent differentiation capacity to WT myoblasts. Scale bar, 200 μm. Graph shows that MHC expression is equivalent between WT and *MCK*-*Cre*/*Xrcc1*^flox/flox^ myoblasts differentiated for 72 h. (**h**) The XRCC1 protein expression profile is similar between WT and *MCK*-*Cre*/*Xrcc1*^flox/flox^ myoblasts differentiated for 72 h.

In addition to acting as a gene expression regulatory event, the loss of XRCC1 in cycling myoblasts may also lead to the accumulation of DNA strand breaks, a genotoxic phenotype that could preclude myoblasts from engaging the differentiation program. However, the doubling time of *Xrcc1*-deleted myoblast cultures was similar to wild-type cells, suggesting that loss of XRCC1 before the onset of differentiation was not a significant impediment for myoblast growth and survival (Figure 5a and b). Cycling *Xrcc1*-deleted myoblasts display a moderate increase in the frequency of DNA strand breakage (as measured with comet assay); yet the transient DNA damage that characterizes normal differentiation is dramatically enhanced in *Xrcc1*-deleted myoblasts. Here, >90% of all *Xrcc1*-deleted myoblasts accumulate extensive DNA damage compared with <40% wild-type myoblasts, which resolve the transient breaks as the differentiation program proceeds (Figure 5c and Supplementary Figure S5a and b). To confirm the differentiation-specific function of XRCC1 (and the origin of the muscle phenotype in the gene-targeted mice), we performed temporal excision experiments in *Xrcc1*^flox/flox^ myoblasts, where *Xrcc1* was deleted following the induction of differentiation. Cre-ad infection of *Xrcc1*^flox/flox^ myoblasts at 3 and 6 h post-low-serum exposure led to a complete block in differentiation, similar to the results obtained for shRNA targeting of the *Xrcc1* gene and *Cre*-mediated deletion of *Xrcc1* in cycling myoblasts (Figure 5d). This experiment confirmed the temporally sensitive role of XRCC1 in stabilizing the differentiation phenotype. Cre-ad addition at 12–48 h post-low-serum induction did not impair formation of myosin heavy chain-positive myotubes in *Xrcc1*^flox/flox^ myoblast cultures, suggesting that loss of XRCC1 at later stages was inconsequential for completion of the differentiation program (Figure 5e), whereas early deletion of XRCC1 shows significant loss of fusion, as well as overall myosin heavy chain protein expression (Figure 5d and f). In addition, flox-flox myocyte/post-myoblast-specific deletion of *Xrcc1* via the generation of *MCK*-*Cre*/ *Xrcc1*^flox/flox^ mice had no observable impact on muscle development (these animals were born with normal Mendelian ratios), whereas primary myoblasts derived from the *MCK*-*Cre*/ *Xrcc1*^flox/flox^ strain displayed the conventional differentiation response as with wild-type myoblasts (Figure 5g). These myoblasts possess the same XRCC1 protein expression profile as wild-type primary myoblasts differentiated up to 72 h (Figure 5h).

## Discussion

Taken together, these results demonstrate that the temporal deployment of the BER-related DNA repair mechanisms (as exemplified by XRCC1) is essential for muscle cell differentiation. Clearly, p21 expression is XRCC1-dependent, an observation suggesting that XRCC1 manages a gene induction program that may be applicable to a wide range of cell lineages. Whether XRCC1 directly promotes a muscle-specific differentiation program remains undefined. Prior observations from our laboratory, along with the current study, suggest that certain elements of the skeletal muscle gene expression program are not influenced by caspase-mediated signaling events, such as the induction of myogenin expression [28]. Nevertheless, other muscle-specific genes appear to be responsive to the caspase/CAD/XRCC1 circuit, including Mef2c, MCK and myosin heavy chain. Identifying the range of XRCC1-responsive genes in a differentiating myoblast will clarify to what extent XRCC1 is a general versus lineage-specific differentiation cue.

XRCC1 is also a component of the backup NHEJ (b-NHEJ) DNA repair pathway, which shares many common features with BER including the recruitment of PARP and DNA ligase III to the strand break site [29]. We have not directly ascertained whether the repair machinery is a BER or b-NHEJ mechanism; however, we favor the hypothesis that XRCC1 is scaffolding a BER repair factory. This supposition is based on the nature of the strand breaks that is predominant during the differentiation program. For example, we have observed that differentiating myoblasts are readily labeled via ISNT using a DNA polymerase, a process that will preferentially label a strand break/nick rather than a double-strand break. In addition, our COMET analysis on differentiating myoblasts displays a qualitatively different electrophoresis profile compared with myoblasts engaging true apoptosis, a cell fate that is characterized by double-strand breaks. Although these measures are not conclusive, they strongly suggest that XRCC1 is participating in a BER mechanism rather than a b-NHEJ repair pathway.

The prevalence of DNA strand breaks and XRCC1 repair foci in early-differentiating muscle cells suggests that this regulated form of damage may target a large number of gene induction events; what remains unknown is whether this targeted break/repair mechanism may also repress gene expression at discrete loci. Given that caspase-mediated signaling is a broadly conserved inductive cue for differentiation, a reasonable supposition is that caspase-driven DNA damage/XRCC1-mediated repair may act as an essential genomic reprogramming event in many cell lineages [23, 30, 31], including gene induction and gene repression. Our observations establish an XRCC1-BER-mediated repair mechanism during differentiation; whether other unrelated or unknown factors assist this process will require future investigations.

## Materials and Methods

### Mice and *in vivo* procedures

All transgenic mice were housed and treated at the University of Ottawa Animal Care and Veterinary Services. Mice used in our studies were housed and cared for according to the Canadian Council on Animal Care guidelines and University of Ottawa Animal Care Committee protocols. *Xrcc1*^flox/flox^ mouse strain was obtained from Dr McKinnon [21]. *Myf5*-*Cre* and *MCK*-*Cre* mouse strains were obtained from Dr Rudnicki [23].

Transgenic pups and their wild-type littermates were taken shortly after birth, post natal<1 day. Hindlimb muscle is extracted and frozen in microfuge tubes at −20 °C until further experiments are performed. Whole-pup bodies were placed in optimum cutting temperature gel-filled embedding molds (Polysciences Inc.) and then flash-frozen in liquid nitrogen to preserve for sectioning and staining. All sectioning and hematoxylin and eosin and Masson’s Trichrome staining were performed by the University of Ottawa, Department of Pathology and Laboratory Medicine, Morphology Unit Lab.

### Cell culture growth and differentiation protocols

For primary myoblast isolations, surgeries were performed as described previously [11]. Briefly, skeletal muscle from hindlimbs is extracted and finely cut with scissors until sufficiently liquefied isolate is achieved. The liquefied muscle chunks are then cultured in a dispase/collagenase solution (dispase II, Roche, New York City, NY, USA, and 1% collagenase (reconstituted in sterile phosphate-buffered saline (PBS)) with 2.5 mM CaCl_2_). Several rounds of trituration and low-speed centrifugation followed by resuspension in Hams’s F-10 Enriched growth media (Ham’s F-10 medium from Gibco Life Technologies, Waltham, MA, USA, 20% fetal bovine serum, 2% penicillin-streptomycin (Pen-Strep), 0.6 μg/ml Fungizone and 2.5 μg/ml βFGF) will yield a population of primary myoblasts sufficiently free of any cross-contaminating tissue remnants. The cells were maintained by replacing the media every 48 h. The cells were allowed to grow on collagen-coated plastic plates (2 ml of rat-tail collagen, Roche, containing 0.2% acetic acid was coated onto tissue culture plates). Ham’s F-10 media is replenished every 48 h to maintain high nutrient content in the solution to maximize the growing potential of primary myoblasts and prevent unaccounted for differentiation. Primary myoblasts are passaged before reaching 70% confluence in the growth dish to further eliminate the chance of spontaneous differentiation. Differentiation was induced in primary myoblast cells by replacing the complete growth media with primary differentiation media (DMEM containing 5% horse serum and 2% Pen-Strep). Cells were collected by scrapping off the plates and centrifugation to pellet cells at the predetermined time points, or differentiation media was changed every 24 h until time point is reached.

### Cell culture shRNA, ISNT, adenovirus and caspase inhibition protocols

Target sequences of shRNA were cloned into the appropriate vector (TRC mouse shRNA individual clone lentiviral pLKO.1 targeted to *Xrcc1*, Open Biosystems/Thermo Scientific, Waltham, MA, USA, pCMV-dsRed-Express Vector, ClontechMountain View, CA, USA, or nonsilencing pGIPZ (shNEG), Open Biosystems/Thermo Scientific) and the plasmid DNA was amplified by culturing the *Escherichia coli* engineered to express each plasmid in antibiotic media containing either Ampicillin or Kanamycin (40 μg/ml in LB broth). After colony expansion the bacterial plasmid DNA was purified using HiSpeed Plasmid Midi Kit from Qiagen, Valencia, CA, USA. The purified plasmid DNA was verified for size by performing a restriction digestion using the restriction enzymes *Bam*HI and *Cla*I. The digests were separated on 1% agarose gels (plus 0.1 μg/ml ethydium bromide) to check that the DNA fragments were of the appropriate size as predicted from the restriction map for the plasmid of interest. One day before transfection, C2C12 myoblast cells were cultured in 35-mm tissue culture plates in Pen-Strep-free growth media to a confluence of 50–60%. The cells were rinsed with 1× OptiMEM I reduced serum media (Gibco Life Technologies) before transfection. The shRNA was prepared by diluting 16 μg/ml DNA per condition in OptiMEM. Similarly, the Lipofectamine 2000 was diluted to 4% in Opti-Mem. The solutions were allowed to incubate for 5 min at room temperature. The Lipofectamine 2000 solution was then gently mixed into the shRNA solution. The Lipofectamine–shRNA solution was allowed to incubate for 20 min at room temperature (RT). Next, 500 μl of the transfection solution was added to each plate and incubated at 37 °C for 3 h. After incubation, 1 ml of antibiotic-free growth media was added to each plate and the cells were incubated for a further 12–18 h. At this point, cell cultures were either induced to differentiate using low-serum media or collected for growth condition.

To perform* *ISNT, C2C12 mouse myoblasts were plated onto ultraviolet-irradiated glass coverslips in 35-mm tissue culture dishes at a concentration of 0.5×10^6^ cells/plate. One plate was prepared for each experimental condition, plus one DNaseI-positive control plate for each experimental parameter. The cells were allowed to grow to confluence in a 37 °C incubator. Before initiating the* *INST assay, the 10× ISNT reaction buffer (0.5 m TRIS-HCl, pH 7.9, 50 mM MgCl_2_ and 100 mM β-mercaptoethanol) and ISNT reaction mix (1× ISNT reaction buffer, 10 μM dNTP (1 mM dCTP, dATP and dGTP), 1 μM dTTP, 1 μM digoxigenin (DIG)-II-dUTP, 0.1% DNA Polymerase I, New England Biolabs, Whitby, ON, Canada) were prepared. Positive control plates were washed in 1× PBS. Next, the coverslips were treated with 200 μl of DNaseI treatment mix (1% DNaseI, 1× DNase buffer) and incubated for 10 min at room temperature. After incubation, the coverslips were washed two times in 1× PBS. The DNaseI control coverslip and experimental condition coverslips were all rinsed with 1× ISNT reaction buffer (made by diluting the 10× reaction buffer 1:10). After rinsing, each coverslip was incubated with 200 μl of ISNT reaction mix and incubated at 37 °C for 45 min with gentle agitation. After 45 min, the coverslips were washed two times in 1× PBS and then immunostained using a mouse anti-digoxigenin (DIG) primary antibody at a concentration of 1:500 in 3% BSA for 1 h at RT.

Adenovirus treatment protocols were used as previously described [23]. Briefly, primary myoblasts isolated from *Xrcc1*^flox/flox^ mice are cultured on collagen-coated plates until 50% confluence is reached. Media is removed and cells are washed with PBS. Cre-ad at multiplicity of infection 10 or Ctl-ad are added to warm reduced serum OptiMEM media. Cre-ad or Ctl-ad media is then added to myoblast plates and allowed to incubate for 1 h at 37 °C, followed by addition of regular Ham’s F-10-enriched growth media, and was left overnight for virus to infect close to 100% cells on plate. Next day, we collect samples for growth or initiate normal differentiation time course protocol described above.

For Caspase 3 inhibition, cultured myoblasts were pre-treated with either 15 μM z.DEVD-FMK (DEVD) from BioVision or 15 μm dimethyl sulphoxide (DMSO) from Sigma-Aldrich (Oakville ON, Canada) for 2 h at 37 °C. After pre-treatment, the cells were induced to differentiate using low-serum media or continued in growth media both containing 15 μM DEVD or DMSO as a vehicle only control. The inhibition or control media was changed every 48 h until the end of the time course. The cells were collected at the predetermined time points and analyzed as described.

### Single-cell gel electrophoresis assay (COMET assay)

Primary myoblast cells from *Xrcc1*^flox/flox^ mice were grown on a 10-cm tissue culture plate, treated with either Ctl-ad or Cre-ad as described above, collected in freezing media and frozen at −80 °C. For the comet assay, the cells were thawed in 37 °C water bath and transferred to growth media. The thawed cells were then centrifuged at 720 *g* for 5 min. The media was then removed and the cells were resuspended in ice-cold 1× PBS. At this point, the cells were ready to proceed using the Comet Assay Reagent kit for Single Cell Gel Electrophoresis from Trevigen, Gaithersburg, MD, USA. During preparation for the comet assay, the Lysis Solution (included in kit) was chilled on ice for at least 20 min before use. Next, the low-melting-point agarose (LMA) was melted by submerging in a beaker of boiling water for 5 min with the cap loosened. The agarose was maintained in a liquid state by transferring it to a 37 °C water bath. The LMA was allowed to cool at 37 °C for at least 20 min before use. Cell samples, at a concentration of 1×10^5^ cells per ml, were combined with LMA at a ratio of 1:10 cells:LMA and immediately pipetted onto labeled Gelbond film strip (agarose gel support medium) Lonza, ME, USA, in 75-μl aliquots. Cells were applied to the hydrophilic side of the film to ensure that the sample spreads evenly into a circle of ~25 mm in diameter. Each film was placed flat at 4 °C in the dark for 10 min or until the gel solidified. The slide was then immersed in pre-chilled lysis solution and incubated at 4 °C for 45 min. After incubation, the lysis solution was removed and the slide was immersed in freshly prepared alkaline solution pH>13 (6 g NaOH, 250 μl 200 mM EDTA, dH_2_O) for 45 min in the dark. The slide was removed from the alkaline solution and transferred to a horizontal electrophoresis apparatus where it was placed equidistant from each electrode in alkaline electrophoresis solution (12 g l^−1^ NaOH, 1 mM EDTA pH 8). Temperature fluctuations were minimized in the non-buffered system by running the electrophoresis at 4 °C in walk in refrigerator. The voltage was set to 30 V for 30 min at constant amperage of 300 mA. Following electrophoresis, the slide was rinsed several times in dH_2_O and then immersed in 70% ethanol to fix. Next, the slide was dried in an air-tight container containing desiccant overnight at room temperature. Once dry, the slide was stained by submerging in SYBR Green stain at 1:10 000 in TE buffer pH 7.5 for 5 min. The slide was then removed and allowed to air-dry. The dry slides were mounted with coverslips and the cells were visualized using fluorescence microscopy using the green fluorescent protein channel (494 nm) at ×20 magnification. Tail length (in μm) for each nucleus is measured using the ImageJ software and is bin-sorted into short (green), medium-short (yellow), long (orange) or extra-long (red) bar graphs.

### Chromatin immunoprecipitation

Hundred million C2C12 cells were collected per time point and fixed in 1% formaldehyde-DMEM solution for 5 min. Arrest of fixation and PBS washing are then performed, and cells are scrapped, pelleted and stored at −80 °C until ready for lysis and shearing. We used the Enzymatic shearing kit from Active Motif (Carlsbad, CA, USA, ChIP-IT Express Enzymatic Cat# 53009) with minor modifications to the procedure. Briefly, we follow instructions in cell lysis using hand-held dounce homogenizer. We enzymatically shear the samples at 37 °C for 10 min with periodic full-speed vortex as instructed. We perform the immunoprecipitation using the amounts indicated for higher volume reactions in the instruction manual, with one major alteration: we use 5 μg of antibody per sample instead of the indicated 1–3 μg. All volumes are adjusted to 200 μl and left on rotators at 4 °C overnight. The following day we proceed to washing the beads, eluting the chromatin and reverse-crosslinking, as described in the manual with one major alteration: we perform the reverse-crosslinking for 4 h at 65 °C and always continue through the proteinase K treatment afterward without storing the samples between steps. The resulting samples (XRCC1-IP, RNApol II-IP, IgG-IP and input DNA samples for each time point) can be used to perform end point PCR at this stage, or the samples can be purified using gDNA purification kits to yield qPCR or Chip-sequencing ready samples. We perform p21 promoter region PCR using primers previously published [11]. EF1alpha primers used for positive control of ChIP is provided in the Active Motif Kit. For Caspase 3 inhibition, we perform DEVD-fmk and DMSO treatments of cells as described above.

### Immunofluorescence staining and microscopy

Cells were cultured on 25-mm coverslips in 35-mm plates and exposed to the described experimental conditions. At appropriate time points, the cell plates were place on ice and the media was aspirated and replaced with ice-cold 1×PBS. The plates were washed a further two times with PBS. The cells were fixed in 90% methanol for 8 min on ice. The fixative was removed and the cells were washed with PBS for two times. Next, the cells were covered with PBS and the plates were wrapped with Parafilm and stored at 4 °C until a time when all time points had been fixed. The plates were tranferred to RT and blocked for 24 h in 3% fetal bovine serum. After blocking, the cells were incubated in primary antibody (DNA ligase IV (H-300) rabbit polyclonal IgG (200 μg ml^−1^), Santa Cruz Biotechnology, Dallas, TX, USA, 1:00, Anti-XRCC1 antibody produced in rabbit (1 mg ml^−1^), Sigma-Aldrich, 1:100, Anti-Myosin Heavy Chain (MF20) mouse IgG, Developmental Studies Hybridoma Bank, Iowa City, IA, USA, 1:50) made up in blocking solution for 24 h at 4 °C. After primary antibody incubation, the cells were washed three times in PBS and incubated in secondary antibody (2 mg ml^−1^ Alexa Fluor 488 goat anti-rabbit IgG (H+L), Invitrogen, CA, USA, 1:500, 2 mg ml^−1^ Alexa Fluor 594 goat anti-rabbit IgG (H+L), Invitrogen, 1:500, 2 mg ml^−1^ Alexa Fluor 594 goat anti-mouse IgG (H+L), Invitrogen, 1:500, AP129F Donkey anti-mouse IgG FLUOR, Chemicon International, CA, USA, 1:500) diluted in PBS for 1 h (RT) to 24 h (4 °C). After incubation, the cells were washed 2× in PBS and 1× in dH_2_O and then counterstained with DNA-specific DAPI, Sigma-Aldrich, made up in dH_2_O (1:10 000) for 10 min at RT. After incubation, the cells were washed two times in PBS and one time in dH_2_O. The coverslips were mounted on microscope slides using Dako Fluorescent Mounting Medium (Dako, Burlington, ON, Canada) and visualized using a Zeiss Observer Z1 inverted fluorescence microscope (Carl Zeiss, North York, ON, Canada). All images are developed using the AxioVision 4.8 software. The quantification of XRCC1 foci was performed by manually visualizing and counting the highest-intensity foci within each individual nucleus and recording the number of foci/nucleus for a minimum of 50 nuclei per experimental condition.

### RNA extraction and PCR

Cells from C2C12 and primary myoblast cultures were washed with ice-cold PBS and lysed using TRIzol reagent. Appropriate volume of chloroform is added to the lysates, followed by the samples being vortexed, and was then allowed to incubate for 3 min at RT after which time they were centrifuged at 10 000 *g* for 10 min at 4 °C to separate the phases. The top phase was removed to a fresh, labeled, RNase-free tube. Appropriate amount of isopropanol was added to each sample and the tubes were gently inverted to mix. The tubes were rocked on a nunator for 20 min at RT, after which time the RNA was pelleted by centrifugation at 10 000* g* for 15 min at 4 °C. The supernatant was discarded and the pellet was washed in 500 μl 70% ethanol in dimethyl pyrocarbonate containing sterile water) and re-centrifuged for 5 min at 7 500 *g* at 4 °C. The supernatant was discarded and the RNA pellet was allowed to air-dry in the fume hood. Once dry, the RNA pellet was resuspended in 80-μl nuclease-free water and allowed to dissolve at 4 °C for 1 h before being stored at −20 °C. First, strand cDNA synthesis was performed using iScript RT Supermix from Bio-Rad (Saint-Laurent, QC, Canada) according to the manufacturer's instructions. All primers used are appended in Supplementary Information section.

### Protein extraction and western blot analysis

Cells from C2C12 and primary myoblast cultures were washed with ice-cold PBS and lysed on ice for 45 min in modified cytoplasmic/nuclear fractionation NE-PER extraction kit from Thermo Scientific. Immunoblot analyses were performed as described previously by using the following antibodies: mouse monoclonal XRCC1 antibody from Abcam (Cambridge, UK, ab1838, 1:1 000), myosin heavy chain and tubulin from Development Studies Hybridoma Bank (MF20, Tub 1:100) and GAPDH from Cell Signaling (Danvers, MA, USA, #2118, 1:2 000). Probe analysis and quantification was performed using the ImageJ software.
