# Supplementary material for: Temporal activation of XRCC1-mediated DNA repair is essential for muscle differentiation
Source: Cell Discov. 2016 Jan 12;2:15041–. doi: 10.1038/celldisc.2015.41 (PMC4860966; doi:10.1038/celldisc.2015.41)
Supplement: Supplementary Figure Legends [file celldisc201541-s1.pdf]

SUPPLEMENTARY INFORMATION:

Supplementary Figure S1 NHEJ pathway is not active during early differentiation. (**a**) Proliferating C2C12 muscle cells were induced to differentiate using low serum media for the indicated time period and were then fixed and stained for DNA Damage repair proteins DNA Ligase4, p-ATM, p-H2AX, 53BP1 and XRCC1. Scale bar, 20µm. (**b**) *Xrcc1* knockout myoblasts fail to fuse into myotubes. *Xrcc1*$^{flox/flox}$ primary myoblast induced to differentiate for 72hr then stained for Myosin Heavy Chain (red) and counterstained with DAPI (blue). (**c**) *Xrcc1* knockout cells have significantly reduced fusion potential, resulting in the inability to form myotubes, as determined by two-tailed student t-test analysis with ***P*-value<0.025

Supplementary Figure S2 shRNA mediated loss of *Xrcc1* impedes myoblast differentiation. (**a**) shRNA mediated knock down of *Xrcc1* in differentiating C2C12 muscle cells impairs the formation of myotubes. Proliferating C2C12 myoblast cells were co-transfected with dsRED plasmid and shRNA directed against *Xrcc1* or a scrambled shRNA negative control plasmid 24hr prior to being induced to differentiate using low serum media. (**b**) Transfection efficiency is similar between control scrambled shRNA and *Xrcc1* targeted shRNA. (**c**) The number of Myosin Heavy Chain positive cells is significantly reduced in shRNA mediated *Xrcc1* knockdown cells, as measured by immunofluorescence. Statistical significance determined by two-tailed student t-test analysis with ****P*-value<0.01 ***P*-value<0.025

Supplementary Figure S3 XRCC1 PCR, Genotyping, and Myosin heavy chain (MHC) protein expression. (**a**) Primary myoblast differentiation time course PCR for *Xrcc1*, *MHC-IIb* and *Gapdh* for control. (**b**) Adult and Embryonic MHC protein expression from WT and cKO hind leg proteins extraction from PN<1day pups, n=2 per genotype. (**c**) Genotyping PCR for *Xrcc1*$^{flox/flox}$ transgene and (**d**) *Cre* transgene from genomic DNA isolated from pup ear clips.

Supplementary Figure S4 Wild type and *Xrcc1* conditional gene targeted phenotypes. (**a**) WT and *Xrcc1* conditional deletion (*Myf5*-Cre/*Xrcc1*$^{flox/flox}$ genotype) pups PN<1day. (**b**) Diaphragm cross sections from WT and cKO pups stained with Masson's Trichrome solutions.

Supplementary Figure S5 *Xrcc1* gene deletion causes accumulation of damage. (**a**) Single cell gel electrophoresis (Comet assay) was performed on differentiating *Xrcc1*$^{flox/flox}$ primary myoblast cells treated with either *Cre*-adenovirus or control-adenovirus. Following electrophoresis, cells were stained with SYBR green and visualized to assess the length of migration of DNA from the nucleus. (**b**) The comet tails were quantified based on tail length (in μm) using ImageJ software and bin sorted into short (green), medium-short (yellow), long (orange) or extra-long (red) tail lengths. Results show increased and persistent DNA damage in *Xrcc1* knockdown cells while control cells exhibit damage early in differentiation that is resolved over the differentiation time course (a statistically significant decrease in the number of short length tails was observed between the 0hr and 12hr time points). Images are representative from n=3 for each experimental condition, minimum number of cells counted = 50 for each condition. Scale bar, 50μm.

PCR Primers Table and qPCR conditions

qPCR cycling conditions: Ta=59C, #cyc=40, SYBR-green Supermix

Instrument Used: Illumina Eco Real-time PCR

Software used: Eco Software v4.1.2.0 and Microsoft Excel 2010 professional Edition

Gene Name	Forward Primer	Reverse Primer
MHC IIb	5’-GGAGAAGAGCGAGCTGAAGA-3’	5’-GGAAAACTCGCCTGACTCTG-3’
Em MHC	5’-GGAGAAGCTCGTCACTTTGG-3’	5’-CCAGTGTCAGCTCAAGGTCA-3’
GAPDH	5’- GACATGCCGCCTGGAGAAAC-3’	5’- AGCCCAGGATGCCCTTTAGT-3’
MyoD	5’-GCTACGACACCGCCTACTAC-3’	5’-GGTCTGGGTTCCCTGTTCTG-3’
Myogenin	5’-ACTCCCTTACGTCCATCGTG-3’	5’- CAGGACAGCCCCACTTAAA-3’
MEF2c	5’-CGGTGTCGTCAGTTGTATGG-3’	5’-TGCAGTAGATATGCGGCTTG-3’
MCK	5’-CATGGAGAAGGGAGGCAATA-3’	5’-GACGAAGGCGAGTGAGAATC-3’
β-actin	5’-AAGGAAGGCTGGAAAAGAGC-3’	5’-AAATCGGCGTGACATCAAA-3’
