# Supplementary figures and images for: Temporal activation of XRCC1-mediated DNA repair is essential for muscle differentiation
Source: Cell Discov. 2016 Jan 12;2:15041–. doi: 10.1038/celldisc.2015.41 (PMC4860966; doi:10.1038/celldisc.2015.41)

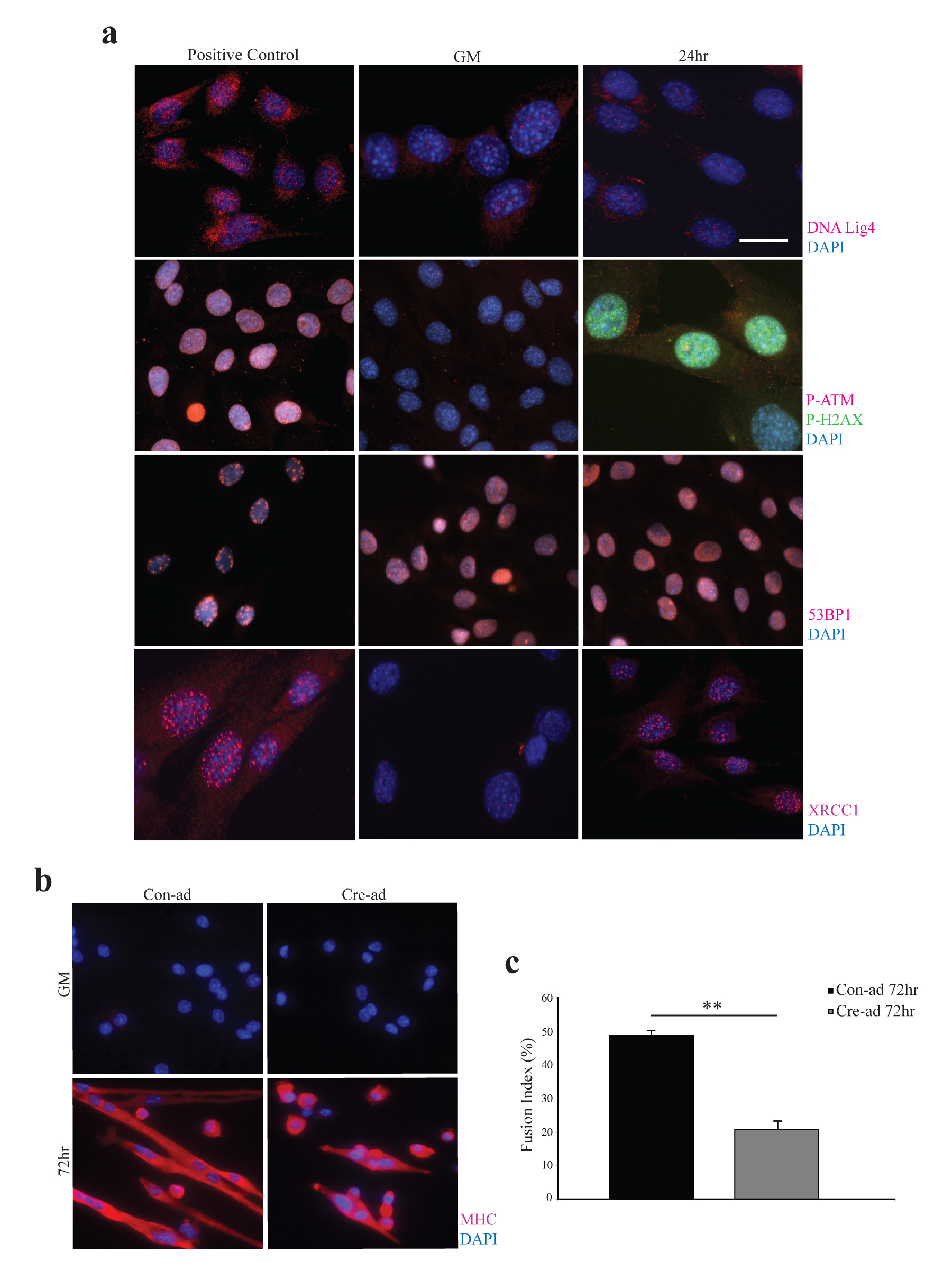

Supplement: Supplementary Figure S1 [file celldisc201541-s2.jpg]

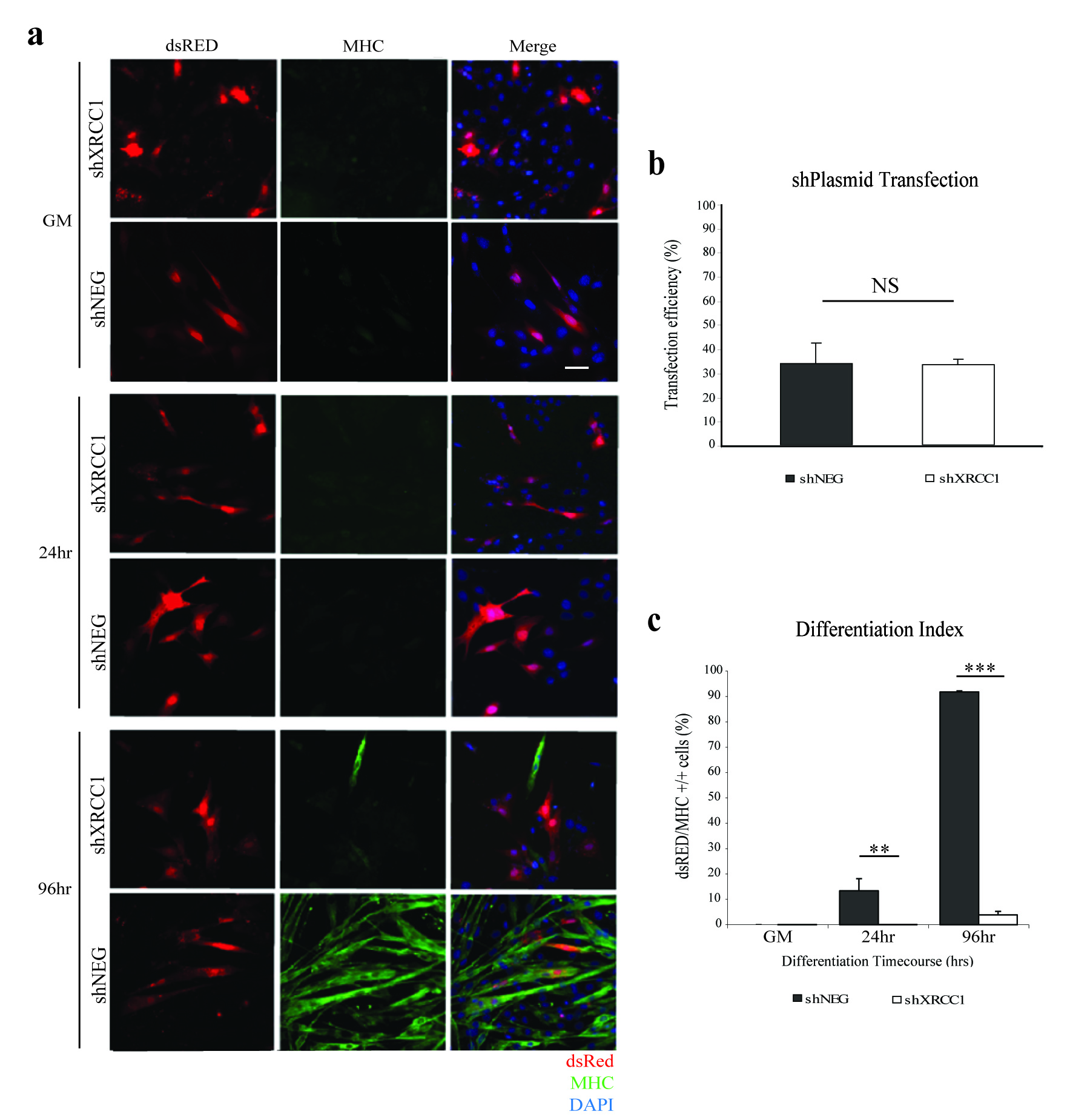

Supplement: Supplementary Figure S2 [file celldisc201541-s3.jpg]

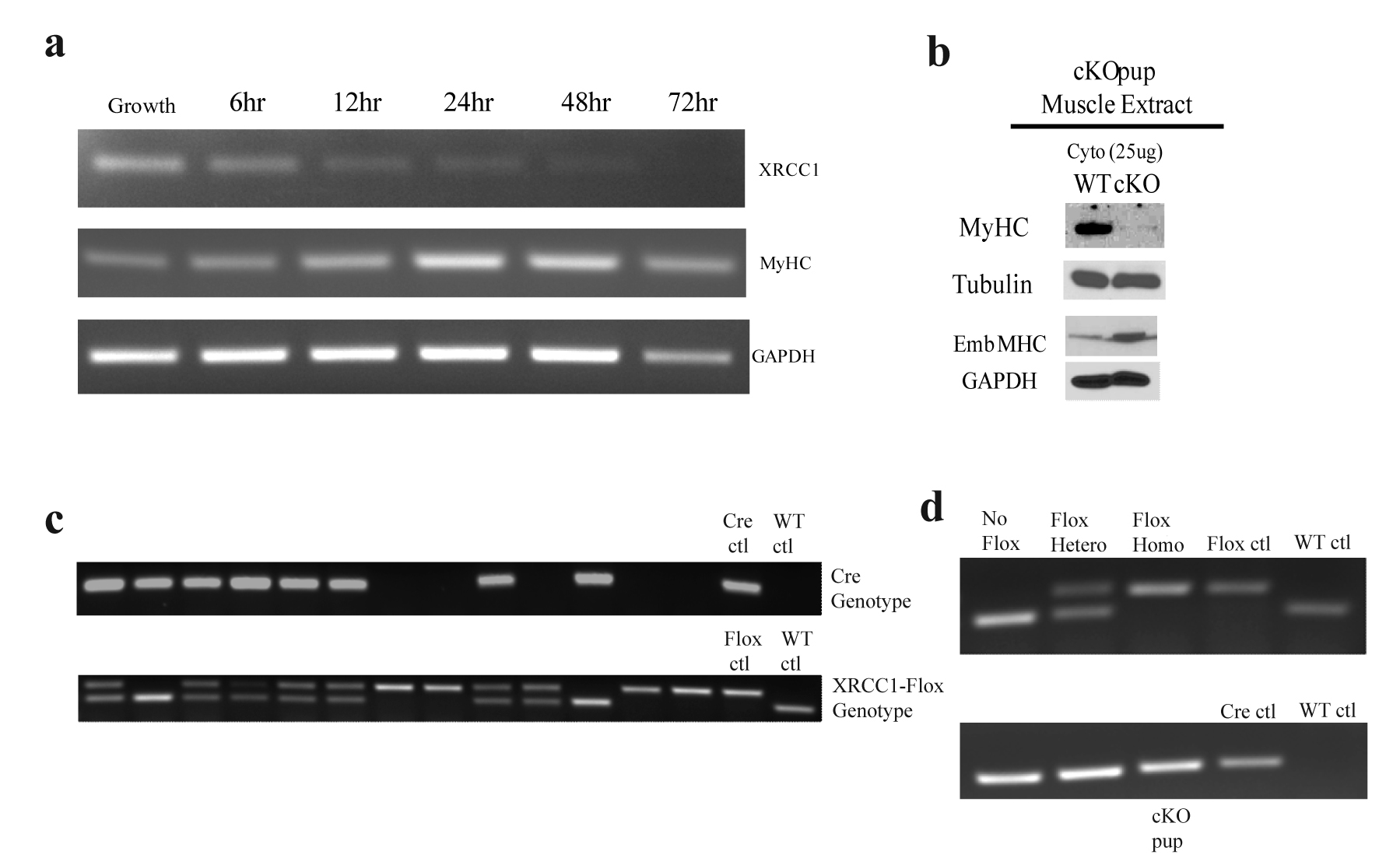

Supplement: Supplementary Figure S3 [file celldisc201541-s4.jpg]

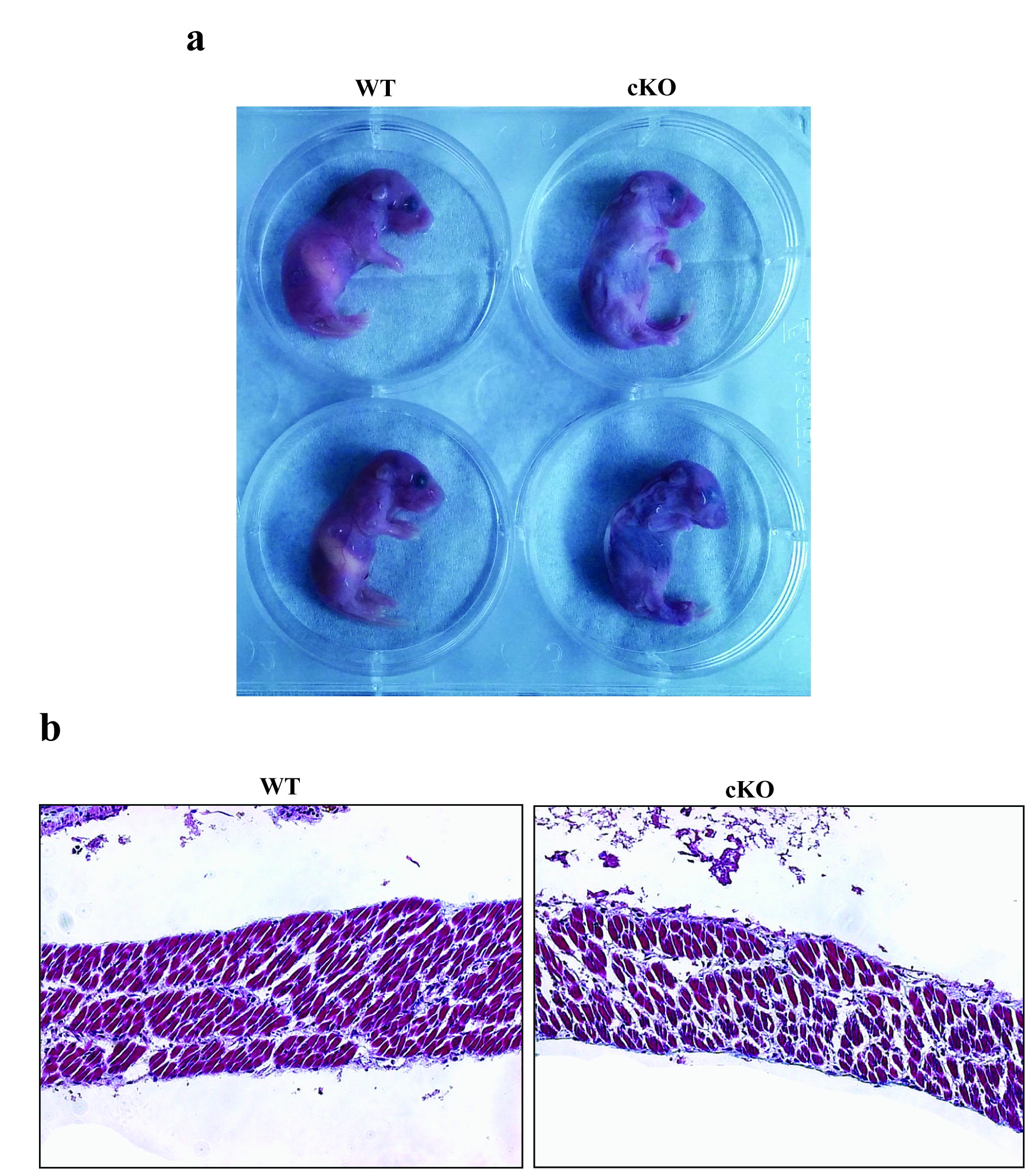

Supplement: Supplementary Figure S4 [file celldisc201541-s5.jpg]

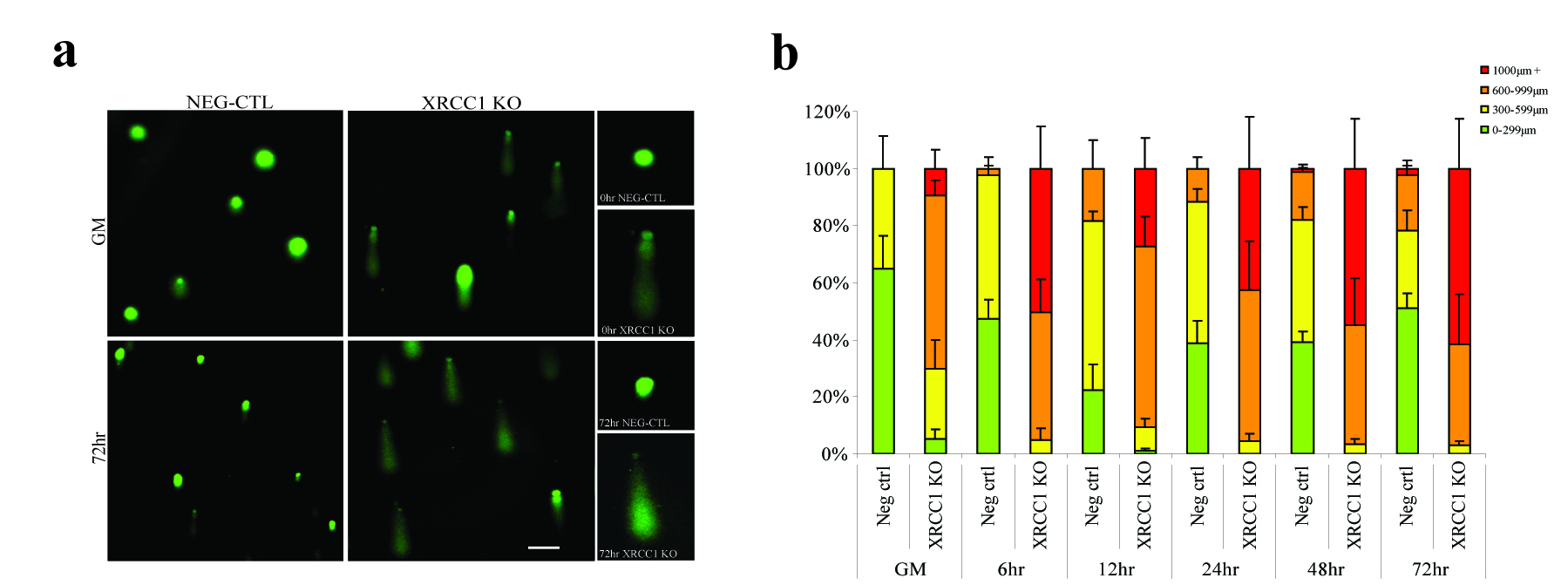

Supplement: Supplementary Figure S5 [file celldisc201541-s6.jpg]
